# Using parent-offspring pairs and trios to estimate indirect genetic effects in education

**DOI:** 10.1002/gepi.22554

**Published:** 2024-03-12

**Authors:** Victória Trindade Pons, Annique Claringbould, Priscilla Kamphuis, Albertine J. Oldehinkel, Hanna M. van Loo

**Affiliations:** 1 Department of Psychiatry, University Medical Center Groningen, University of Groningen, Groningen, The Netherlands; 2 Department of Genetics, University Medical Center Groningen, University of Groningen, Groningen, The Netherlands; 3 Structural & Computational Biology Unit, European Molecular Biology Laboratory, Heidelberg, Germany; 4 Department of Hematology, University Medical Center Groningen, University of Groningen, Groningen, The Netherlands

**Keywords:** cultural transmission, dynastic effects, educational attainment, genetic nurture, indirect genetic effects, intergenerational transmission

## Abstract

We investigated indirect genetic effects (IGEs), also known as genetic nurture, in education with a novel approach that uses phased data to include parent-offspring pairs in the transmitted/nontransmitted study design. This method increases the power to detect IGEs, enhances the generalizability of the findings, and allows for the study of effects by parent-of-origin. We validated and applied this method in a family-based subsample of adolescents and adults from the Lifelines Cohort Study in the Netherlands (*N* = 6147), using the latest genome-wide association study data on educational attainment to construct polygenic scores (PGS). Our results indicated that IGEs play a role in education outcomes in the Netherlands: we found significant associations of the nontransmitted PGS with secondary school level in youth between 13 and 24 years old as well as with education attainment and years of education in adults over 25 years old (*β* = 0.14, 0.17 and 0.26, respectively), with tentative evidence for larger maternal IGEs. In conclusion, we replicated previous findings and showed that including parent-offspring pairs in addition to trios in the transmitted/nontransmitted design can benefit future studies of parental IGEs in a wide range of outcomes.

## 1 ∣ BACKGROUND

Parental genotypes impact their offspring directly through genetic transmission, but may also have an effect that is mediated by the environment. Such indirect genetic effects (IGEs), also known as “genetic nurture” (Kong et al., 2018), are particularly relevant for traits that are influenced by the shared environment within families, such as sociobehavioral and psychiatric traits. Understanding IGEs can provide valuable insights into the interplay between genetics and the environment in shaping these traits, such as the genetic mechanisms underlying cultural transmission and the role of gene-environment correlations. Moreover, accounting for IGEs is needed to accurately estimate direct genetic effects (Morris et al., 2020).

Pioneering studies reported IGEs in education by distinguishing between parental genetic variants that were transmitted and those that were *not* transmitted (i.e., parental-exclusive) to the offspring (Bates et al., 2018; Kong et al., 2018). In these studies, parental transmitted and nontransmitted genotypes were separated and used to compute two polygenic scores (individual-level genetic liability for a trait; PGS) for educational attainment. Next, the authors assessed the relationships between the (non)transmitted PGS and educational outcomes in the offspring. In addition to the expected association of offspring outcomes with the transmitted PGS, the findings also revealed a nonzero effect of the nontransmitted PGS, indicating a genetic component to environmental pathways of education inheritance, such as parental rearing style and socioeconomic status (Bates et al., 2018; Demange et al., 2022; Selzam et al., 2019). These findings have since been replicated and are robust across multiple countries and methods (Cheesman et al., 2020; Howe et al., 2022; Wang et al., 2021; Wu et al., 2021; de Zeeuw et al., 2020).

This study design typically requires genotyping data for offspring and both parents (parent-offspring trios). However, despite the growing amount of family data available in biobanks, the number of parent-offspring trios has remained moderate, and genotyping data is commonly only available for one parent. Solutions to include parent-offspring pairs in these analyses would allow the use of larger samples and improve the generalizability of the findings.

Detection of IGEs on a trait through nontransmitted PGS requires large sample sizes, because it is dependent on the predictive value of the PGSs, which are limited by the power of the study used to train the score (Bruins et al., 2020). Because PGSs only account for a fraction of the total additive genetic variance in most complex traits, large samples are required to ensure sufficient statistical power to detect IGEs.

The inclusion of pairs could also help to address potential selection biases in trio-based studies. As part of their study on IGEs in ADHD, Martin et al. (2023) compared parent-offspring pairs and trios in terms of clinical and demographic variables. They found notable differences in pairs, including lower IQ and parental socioeconomic status, and more severe clinical profiles. These initial findings suggest that a study design that requires both parents to participate in the study may be biased, possibly because families in which both parents choose to participate in a study tend to be more intact and/or less dysfunctional, which may limit the generalizability of results (Yu & Scharf, 2023).

We developed a method that leverages haplotype information to allow the inclusion of parent-offspring pairs in analyses of transmitted and nontransmitted genetic effects. This method also extends opportunities to investigate effects by parent-of-origin, as it allows for the determination of nontransmitted alleles by parent when both the offspring and parents are heterozygous at a locus. To validate this approach, we investigated the concordance between our results and the output of standard software and assessed the internal consistency by simulating parent-offspring pairs out of trios.

Next, we applied this approach to a large family-based sample that includes both parent-offspring pairs and trios. We aimed to validate the method and replicate previous findings of genetic nurture on educational outcomes of adolescents and adults. We also investigated parent-specific IGEs and examined potential demographic differences between parent-offspring pairs and trios in our sample.

## 2 ∣ METHODS

### 2.1 ∣ Sample

We analyze data from Lifelines, a multi-disciplinary prospective population-based three-generation cohort study examining the health and health-related behaviors of 167,729 persons living in the North of the Netherlands. It employs a broad range of procedures to assess biomedical, socio-demographic, behavioral, physical, and psychological factors that contribute to the health and disease of the general population, with a special focus on multimorbidity and complex genetics. A detailed description of the Lifelines Cohort study can be found elsewhere (Sijtsma et al., 2022).

Genotyping data was available for 7020 participants with at least one parent genotyped. Seven hundred and ten participants were excluded because they were under 13 years old and thus had not yet completed their education, and 163 because of missing phenotype data, leaving a total of 6147 offspring.

### 2.2 ∣ Measurements

#### 2.2.1 ∣ Educational outcomes

Educational outcomes were coded based in the Dutch school system. After primary education, Dutch students are grouped into three levels for secondary schooling: prevocational education (VMBO), higher general continued education (HAVO), and preparatory scientific education (VWO). This placement is defined after age 12, informed by teacher's advice and performance in a nationwide standardized test, and is highly predictive of an individual's final educational attainment. As each educational track takes a distinct time to complete, information about final education attainment can be ambiguous under a certain age. Therefore, individuals were grouped into two subsets: youth (age 13–24, *N* = 1883) and adults (age ≥25, *N* = 4264). Education was defined in the youth subset by an ordinal variable with three levels representing secondary education level. Current school level was used for individuals under 17 years old, and highest obtained degree was used to code secondary school level for individuals between 18 and 24 years old (Supplementary Materials). For adults, we used two outcome variables based on highest obtained degree: educational attainment and years of education. Educational attainment was defined as an ordinal variable with three levels: low (primary or prevocational secondary education), middle (higher general continued education or preparatory scientific education), and high (university of applied sciences or research university). This ordinal measure was used to allow for comparison with the youth sample. Years of education was coded as the minimum number of cumulative years required to finish the highest achieved degree, which provided a more fine-grained measure of educational attainment.

#### 2.2.2 ∣ Income

Adult Lifelines participants were asked to report their net household income along with the number of people living on this income. We calculated the household equivalent monthly income using the square root scale method. This method adjusts the household income for the number of people in the household by dividing the household income by the square root of the number of people. The resulting household equivalent monthly income was used to categorize the households into three groups: low (<1100 Euro), intermediate (1100–1899 Euro), and high (≥1900 Euro) income (Klijs et al., 2016).

Parental education years and household income were defined as the mean of both parental phenotypes if data was available for both, or the phenotype of the single available parent otherwise.

### 2.3 ∣ Genotyping data

We used the first data release of the UMCG Genetics Lifelines Initiative (UGLI), which used the Infinium Global Screening Array^®^ (GSA) MultiEthnic Disease Version 1.0 to genotype 38,030 Lifelines participants. This release contained 557,184 markers on autosomal chromosomes that passed standard QC steps in individuals of European ancestry. In brief, monomorphic markers, markers with a high missing rate (>1%), minor allele frequency <5%, or a Hardy-Weinberg *p* value ≤ 1 × 10^−6^ were removed. Samples that had missing rate >1%, contained heterozygosity outliers or were identified as mix-ups were filtered out. Population stratification was examined by a principal components analysis using samples from 1000 Genomes and GoNL projects. More detailed information, including a complete quality control report, can be found online in the Lifelines wiki (http://wiki.lifelines.nl/doku.php?id=ugli).

### 2.4 ∣ Nontransmitted alleles inference

**FIGURE 1:**
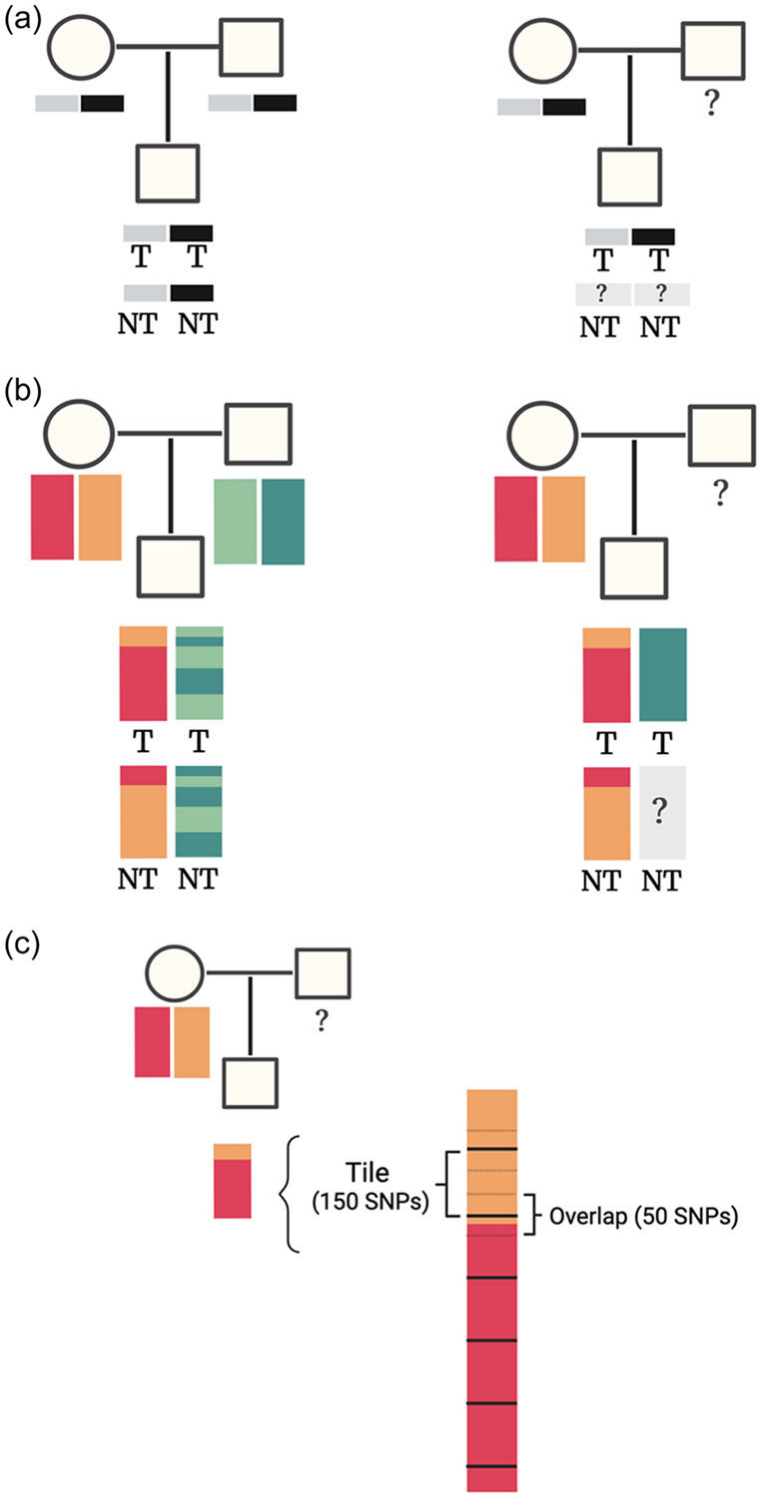
Differentiation of transmitted (T) and nontransmitted (NT) alleles in pedigrees representing an individual with both parents genotyped (parent-offspring trio) and with genotypes missing for one parent (parent-offspring pair). (a) Comparison per SNP: for a single locus, it is possible to determine NT alleles if all genotypes are available. This SNP-by-SNP comparison is used in PseudoCons and in the --tucc option in Plink. However, if the genotype is missing for one of the parents and both the observed parent and offspring are heterozygous for this locus, both alleles could have been inherited from the nonmissing parent. It is therefore impossible to resolve which alleles are NT. (b) Comparison by haplotypes: the haplotypes of the offspring are inherited as a unit from each parent. Crossing-over of genetic material during meiosis result in offspring's haplotypes having different combinations of alleles from the originating parent. We determined NT alleles by approximating identity-by-descent segments: matching segments of DNA that are shared between parents and their offspring without recombination. For parent-offspring pairs, NT alleles could only be determined for the nonmissing parent. (c) To compare haplotypes, every 150 adjacent SNPs in a chromosome were aggregated into tiles. The markers included in each tile overlapped with neighboring tiles by one third, which allowed the identification of recombination spots. Tiles are used to trace the best matching parental haplotype across all the tiles. For each tile, if the overall match between the offspring's haplotype and the best matching parental haplotype is less than 99.8%, the method assumes that there could be recombination and checks the match with the other haplotype from the same parent. Thus, the transmitted alleles are a combination of the overall best matching haplotype for most tiles and the recombination haplotype for the tiles that matched better with the other haplotype for that same parent. The nontransmitted alleles are the remaining parental tiles that were not identified in the offspring. Figure created with BioRender.com.

Standard software, such as PseudoCons (Cordell et al., 2004) or the --tucc option in PLINK (Purcell et al., 2007), performs a marker-by-marker comparison to resolve which parental genotypes were not transmitted to the offspring. This comparison is not possible for every marker when one of the parental genotypes is missing (Figure 1a). To enable the inclusion of parent-offspring pairs, we used haplotypes, which are sets of markers that are inherited together on each chromosome. The linkage patterns between loci make it possible to determine the nontransmitted alleles for the observed parents and to distinguish the alleles by parent-of-origin (Figure 1b).

We used SHAPEIT4 (Delaneau et al., 2019) to estimate haplotypes from genotype data (phasing). Then, we compared offspring's haplotypes to the two (pairs) or four (trios) parental haplotypes using tiles, which are blocks of adjacent markers on a chromosome. Tile size of 150 markers was optimal for our genotyping data, as the number of markers in a tile depends on the SNP density measured by the array (Supplementary Materials). For each available parent, the best match between parent and offspring tiles was used to determine which two parental tiles were transmitted to the offspring. To enable identification of crossing-over events, in which the best match may switch from one to the other haplotype of the same parent, we included a third of the markers (50) that overlap with neighboring tiles in each tile (Figure 1c). After determining the transmitted tiles, the remaining parental tiles were written in a separate data set as the nontransmitted alleles. For parent-offspring pairs, the nontransmitted alleles of the unobserved parent were set as missing.

### 2.5 ∣ Imputation

IMPUTE5 (Rubinacci et al., 2020) was used for imputation of transmitted and nontransmitted data sets separately, with 1000 Genomes phase3v5 as reference panel. To circumvent missing nontransmitted data for parent-offspring pairs during the imputation process, we included duplicated haplotype information for each individual. After imputation, duplicated markers, and markers with INFO scores <90% were filtered out, and markers were restrained for the overlap with HapMap3 high-quality markers. Finally, 812,047 variants were available for PGS computation.

### 2.6 ∣ PGS

PGS were computed based on the latest GWAS for educational attainment (EA4) including 765,283 subjects and 10,985,947 SNPs as discovery data set (Okbay et al., 2022). The Lifelines subsample examined within this study is completely independent from the discovery data set. We removed 7507 markers in the MHC region of chromosome 6 (Supplementary Materials) and used the overlap between summary statistics and imputed genotypes which resulted in 809,503 variants to be included in the score. SNP effects were reweighted using LDpred2, a Bayesian method that uses genetic architecture and an ancestry-matched LD reference as priors (Privé et al., 2021). We used the auto model, which estimates these parameters directly from the training data.

For each offspring, PGS were created based on transmitted and nontransmitted data sets. The transmitted PGS (PGS_T_) and nontransmitted PGS (PGS_NT_) were defined as the sum of the PGS based on paternal and maternal transmitted and nontransmitted alleles, respectively. To create PGS_NT_ in parent-offspring pairs, the value of the missing PGS_NT_ was imputed with the mean half PGS of all of the observed parents.

### 2.7 ∣ Statistical analyses

To compare parent-offspring trios and pairs, we used *t* tests for age and years of education, and *χ*^2^ tests for sex, secondary school level, EA, and parental income.

For the method validation, we performed the following analyses:

1. Concordance of the nontransmitted output with PseudoCons: PseudoCons (Cordell et al., 2004) is a software tool to create pseudo controls from pedigree data based on nontransmitted parental genotypes. We compared the assigned nontransmitted alleles for each SNP in parent-offspring trios across the two methods. We computed the percentage of discordant alleles, or mismatches, between the two methods for each parent-offspring trio for each chromosome. We then calculated the mean percentage of discordant alleles per chromosome.
2. Simulating parent-offspring pairs out of trios: We randomly set the genotypes of one parent in five trios as missing, to evaluate if the match between parental and offspring haplotypes was consistent regardless of whether the offspring was included as a trio or a pair. The best match between offspring and parent tiles should be the same for both scenarios, with the only difference being that the nontransmitted haplotype of the unobserved parent is not present when the offspring is included as a pair. This analysis tests if the missing data in pairs affects the output.

Statistical analyses were performed in R version 4.0.3 (R Core Team, 2022). To estimate direct and indirect genetic effects in educational outcomes, we used the ordinal package (Christensen, 2022) to fit cumulative link mixed models for ordinal outcomes and lme4 (Bates et al., 2015) to fit linear mixed models for years of education. PGS_T_ and PGS_NT_ were included in all models as predictors, along with sex, age, and the first 10 principal components. All continuous predictors were z-standardized. Sibling relationships in the data were accounted for by including a random intercept for family identifier.

## 3 ∣ RESULTS

### 3.1 ∣ Sample description and comparison between parent-offspring pairs and trios

The participants in the youth subsample (*N* = 1883) had mean age 19.1 years (SD 3.4). This sample consisted of 1691 (89.8%) parent-offspring pairs and 192 (10.2%) complete parent-offspring trios. There was no evidence for meaningful differences between offspring in trios or pairs for age, sex, or secondary school level. Income and years of education were not different between parents in trios and pairs.

The adult participants (*N* = 4264) had mean age 35.0 years (SD 6.8). This subsample included 3703 (86,8%) parent-offspring pairs and 561 (13.2%) complete trios. Offspring in trios and pairs were not different by sex, education attainment, and years of education. Although some differences between parent-offspring pairs and trios were statistically different (age, parental income, parental years of education), these were not substantial (Table 1).

### 3.2 ∣ Method validation

First, we compared the concordance of our nontransmitted alleles with these produced by PseudoCons in parent-offspring trios. The nontransmitted alleles assigned by PseudoCons and the method presented in this study were rarely mismatched. More than 99.8% of assignments were in agreement between methods for all trios, showing that our haplotype-based method performs with very high similarity to discerning nontransmitted alleles per marker. This means that the nontransmitted PGS for an individual is consistent across both methods, ensuring equal effects in downstream analyses.

Second, we simulated parent-offspring pairs out of trios and compared the match between parental and offspring tiles. For every offspring, we confirmed that the match between parental and offspring haplotypes was consistent regardless of whether the offspring was included as a trio or a pair, which confirmed that our method is not sensitive to the structure of the data and that the missing data in pairs does not affect the output.

### 3.3 ∣ Direct and indirect genetic effects in educational outcomes

**FIGURE 2:**
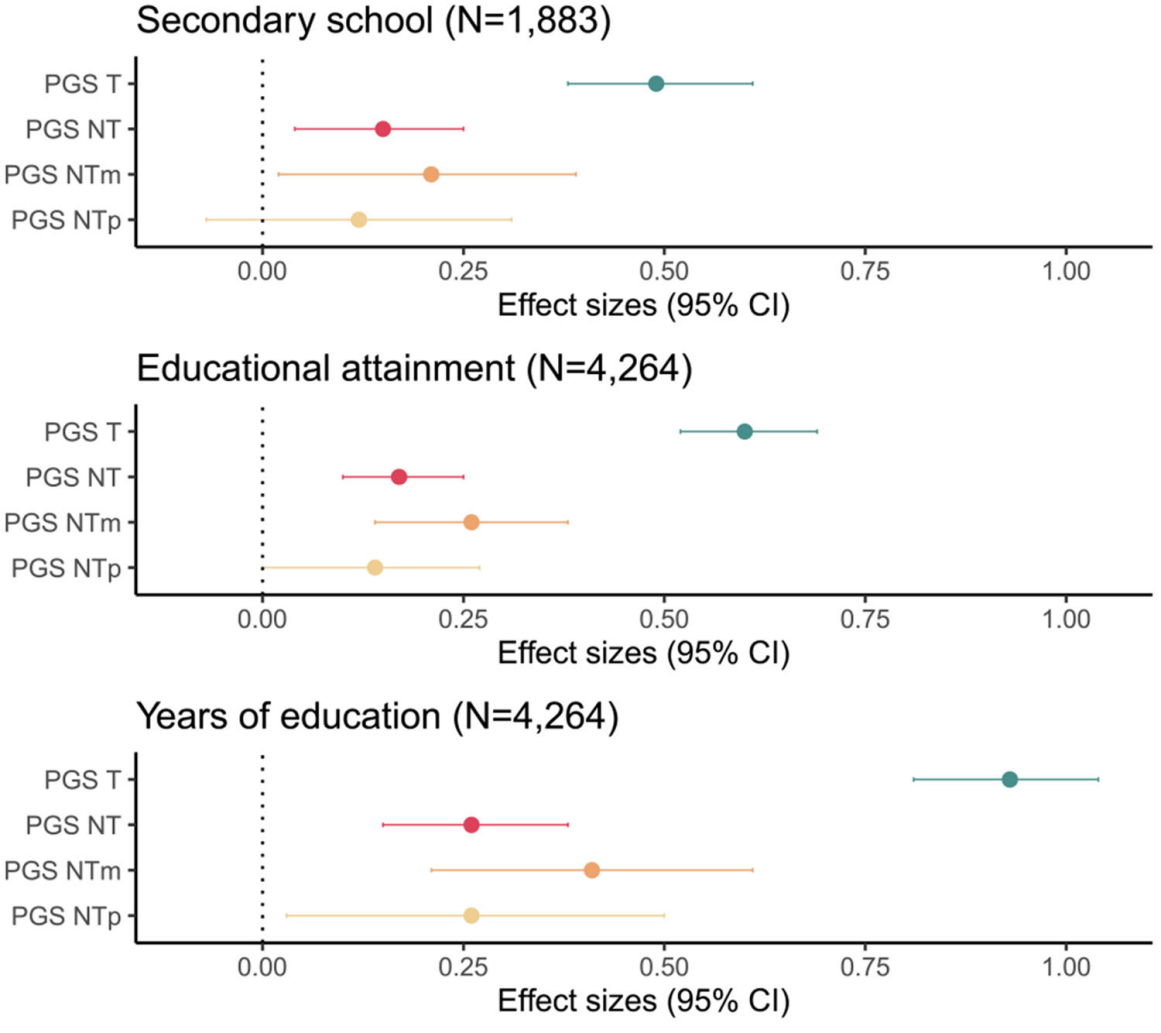
Standardized effect sizes (*β* coefficients) and 95% confidence intervals for full transmitted (PGS_T_) and nontransmitted (PGS_NT_) polygenic scores based on educational attainment, as well as nontransmitted polygenic scores separated by parent (maternal PGS_NTm_ and paternal PGS_NTp_) analyzed in a joint model. The youth sample (ages 13–24) had secondary school level as an ordinal outcome, although the adult sample (ages 25 and above) had educational attainment (ordinal) and years of education (continuous) as outcomes.

As expected, PGS_T_ was associated with all outcomes. PGS_T_ had a greater effect in years of education (*β* = 0.93 [0.81–1.04], *p* < 0.001) as compared with the categorical secondary school level (*β* = 0.49, CI [0.38–0.61], *p* < 0.001) and education attainment (*β* = 0.60, CI [0.52–0.69], *p* < 0.001), as seen in Figure 2. This is likely because years of education is a more precise variable than the ordinal variable and is more similar to the phenotype on which the PGS discovery data set was based.

We also found significant associations of PGS_NT_ with all outcomes: secondary school level in youth (*β* = 0.14, CI [0.04–0.25], *p* < 0.01) and both education attainment (*β* = 0.17, [0.09–0.25], *p* < 0.001) and years of education (*β* = 0.26, 95% CI [0.14–0.38], *p* < 0.001) in adults (Figure 2). This shows that it is possible to detect IGEs in education in a sample with both parent-offspring trios and pairs. Our results yield similar ratios of indirect to direct effects across outcomes: 0.38 for secondary school level, 0.39 for educational attainment, and 0.40 for years of education.

### 3.4 ∣ IGEs by parent-of-origin

Across outcomes, indirect maternal effects were consistently larger than paternal effects, in analyses where PGS_NTp_ and PGS_NTm_ were analyzed jointly (Figure 2) and in separate models (Supporting Information: Table 2). However, given that the confidence intervals for the effect sizes of PGS_NTm_ and PGS_NTp_ overlap, we cannot definitively conclude that there is a difference in magnitude of IGEs by parent (Table 2).

## 4 ∣ DISCUSSION AND CONCLUSIONS

We examined direct and indirect effects of parental genotypes on offspring education through PGS with a novel approach that uses haplotypes to distinguish between transmitted and nontransmitted alleles. This strategy improves upon previous methods by increasing sample size and generalizability, because it allows inclusion of parent-offspring pairs in addition to trios and investigation of effects by parent-of-origin. Our results provide evidence for IGEs in the educational outcomes of adolescents and adults in the Netherlands, with tentative indications of larger maternal than paternal effects.

We found significant associations between nontransmitted PGS and offspring educational outcomes. The magnitude of the indirect effects relative to the direct genetic effects (~0.4) falls within the wide range of ratios reported in previous studies of genetic nurture in education (0.4–0.8). A meta-analysis of eight studies reported a higher ratio based on pooled estimates (0.6), but it is important to note that most included studies used a method that systematically yields higher estimates of indirect effects than the one used in the present study (Wang et al., 2021). Our ratios were highly comparable with two studies that also used the nontransmitted PGS method, Bates et al. (2018) and Kong et al. (2018), but lower than those found in a study conducted by de Zeeuw et al. (2020), who used the same method in adult twins in the Netherlands. Heterogeneity in sample size, statistical analyses, PGS training data, and outcome measures across studies may contribute to the variability observed in indirect-to-direct genetic effects across studies. Overall, our study extends the body of evidence supporting the role of IGEs, emphasizing the complex nature of genetic contributions to the traits impacting educational outcomes.

IGEs may differ across the lifespan, thus detecting them may depend on the educational outcome being examined. Demange et al. (2022) and de Zeeuw et al. (2020) found significant IGEs on educational attainment in Dutch adults but not in academic achievement measured by national test scores (Cito exam) at age 12. In contrast, we detected effects of nontransmitted PGS on the level of secondary school in adolescents. This difference could stem from our increased power to detect IGEs in youth. Another possible reason concerns the educational tracking system in the Netherlands. During primary school, the core curriculum and benchmark levels are, in principle, the same for all children, thus parental influences may only have a modest influence. Thus, when children take the Cito exam at age 12, their scores are primarily affected by their own genetics (direct genetic effects). For secondary education, children enter one of three tracks representing different levels, and the choice for a specific track is known to be partly influenced by social factors (Timmermans et al., 2018). Indeed, in the Netherlands, children whose parents are more highly educated are more likely to enter a higher educational track themselves, regardless of their test performance (van Spijker, 2017). Further investigation, preferably in longitudinal cohorts, is needed to better understand whether and how genetic nurture effects change across age, and how these effects may be moderated by the educational system.

Parent-of-origin analyses aim to investigate whether there is a difference in the magnitude of IGEs between mothers and fathers. In contrast to previous studies that suggested that these effects do not differ by parent in education (Kong et al., 2018; Wang et al., 2021), we found that maternal effects were consistently larger than paternal effects in our study. Although we were unable to determine if this difference was statistically significant, it is possible that it reflects unique characteristics of our sample. Women work on average fewer hours than men in the Netherlands, and the gender gap in work hours in the Netherlands is the largest in Europe (OECD, 2019). Thus, mothers may have a stronger influence on the home environment. This suggests that parental involvement can moderate the indirect effect of parental genes in education. This notion converges with a study in Israel in which the parent–child correlation in education was stronger with the parent who spent more time with the child than with the parent who spent relatively less time parenting (Gould et al., 2020). Future analyses could include information about household composition to explore the impact of varying levels of parental exposure on IGEs.

By using a haplotype-based approach, we were able to increase our statistical power to detect IGEs beyond what would have been possible using trios only. In addition, the inclusion of parent-offspring pairs can improve the representativeness of the sample. When investigating IGEs on attention-deficit hyperactivity disorder, Martin et al. (2023) reported differences between families represented by pairs and trios. In particular, they observed that offspring that were part of pairs had higher levels of hyperactive-impulsive and conduct disorder symptoms, as well as lower IQ, and their parents had lower educational attainment, income, and SES. We also found that parent-offspring pairs showed slightly lower levels of education and income in our sample. Such differences between families represented by trios and pairs probably depends on the outcomes being studied. In general, including pairs increases the likelihood of including alternative family arrangements and families with very high or low parental involvement. In other words, along with increasing the sample size substantially, our approach also improves on previous methods by capturing a broader range of familial environments, thus enhancing the generalizability of the results.

Our study highlights the benefits of including parent-offspring pairs in addition to trios in analyses of genetic nurture, but there are limitations that should be taken into consideration. First, the missing data in pairs can impact the estimates of IGEs: we partly imputed the nontransmitted PGS by using the mean of the nonmissing data, which can result in attenuated effect sizes. Another limitation is that population-level phenomena such as assortative mating and residual population stratification can inflate the effect of the nontransmitted PGS, although the magnitude of this confounding effect is likely to be small (Kong et al., 2018; Shen & Feldman, 2020).

In conclusion, IGEs enhance our understanding of the intergenerational transmission of sociobehavioral traits. Our haplotype-based method to include parent-offspring pairs along with trios in the transmitted/ nontransmitted design offers new opportunities to study these effects in larger and more diverse samples. Our findings suggest that contextual factors such as the educational system and parental involvement may moderate the impact of parental IGEs.

## Supplementary Material

Supplement

## Figures and Tables

**TABLE 1:** Sample characteristics and comparison between parent-offspring trios or pairs within youth and adult subsamples.

	Youth (age range 13–24)				Adults (age range 25–67)			
	Total (*N* = 1883) Mean (SD) or %	Parent-offspring pairs (*N* = 1691) Mean (SD) or %	Parent-offspring trios (*N* = 192) Mean (SD) or %	Comparison	Total (*N* = 4264) Mean (SD) or %	Parent-offspring pairs (*N* = 3703) Mean (SD) or %	Parent-offspring trios (*N* = 561) Mean (SD) or %	Comparison
Age	19.1 (3.4)	19.0 (3.4)	19.5 (3.3)	*t* = 1.95 *p* = 0.05	35.0 (6.8)	35.0 (6.9)	34.1 (5.5)	*t* = −3.56 *p* < 0.001
Sex (female)	60%	60%	58%	*χ*^2^ = 0.32 *p* = 0.57	59.6%	59.4%	61.3%	*χ*^2^ = 0.71 *p* = 0.40
Educational attainment/Secondary school level	25.2/39.1/35.6	25.5/38.8/35.6	22.0/41.6/35.9	*χ*^2^ = 1.03 *p* = 0.59	11.4/42.4/46.1	11.8/42.5/45.5	8.7/41.8/49.3	*χ*^2^ = 5.78 *p* = 0.056
Education years Range 1–20	–	–	–	–	15.8 (3.9)	15.8 (3.9)	16.1 (3.9)	*t* = 1.87 *p* = 0.68
Parental household income	18.4/72.3/9.2	18.8/71.9/9.1	14.5/75.7/9.6	*χ*^2^ = 2.93 *p* = 0.23	12.7/36.7/32.1	13.3/38.5/30.9	8.3/40.6/40.1	*χ*^2^ = 19.51 *p* < 0.001
Parental education years Range 1–20	13.9 (3.4)	13.9 (3.4)	14.0 (3.3)	*t* = 0.44 *p* = 0.06	12.3 (3.5)	12.2 (3.5)	12.9 (3.4)	*t* = 4.53 *p* < 0.001

*Note*: Continuous variables are presented as mean and standard deviation (SD), and percentages are presented for categorical variables. The comparison results are reported using either a chi-squared (*χ*^2^) or *t* test statistic. The sample size for offspring with no siblings in youth was *N* = 1443 and for adults was *N* = 3126.

**TABLE 2:** Main results: effects of transmitted and nontransmitted polygenic scores based on educational attainment.

	*β* (SD)	95% CI	OR	95% CI	*p*
*Youth N = 1883*					
Secondary school					
PGS_T_	0.49 (0.06)	0.38–0.61	1.63	1.45–1.84	<0.001
PGS_NT_					
Both parents	0.15 (0.06)	0.04–0.25	1.16	1.03–1.31	<0.01
Paternal	0.12 (0.09)	−0.07–0.31	1.13	0.95–1.35	0.23
Maternal	0.21 (0.09)	0.02–0.39	1.23	1.03–1.47	0.02
*Adults N = 4264*					
Educational attainment					
PGS_T_	0.60 (0.04)	0.52–0.69	1.82	1.68–1.97	<0.001
PGS_NT_					
Both parents	0.17 (0.04)	0.10–0.25	1.19	1.10–1.28	<0.001
Paternal	0.14 (0.07)	0.00–0.27	1.15	1.00–1.32	0.05
Maternal	0.26 (0.06)	0.14–0.38	1.30	1.15–1.46	<0.002
Years of education					
PGS_T_	0.93 (0.06)	0.81–1.05	–	–	<0.001
PGS_NT_					
Both parents	0.26 (0.06)	0.15–0.38	–	–	<0.001
Paternal	0.26 (0.11)	0.03–0.50	–	–	0.02
Maternal	0.41 (0.10)	0.21–0.61	–	–	<0.001

*Note*: Effect sizes are reported with standardized *β* along with their standard deviation (SD) and odds ratio (OR) for ordinal outcomes with 95% confidence intervals (CI) for full transmitted (PGS_T_) and nontransmitted (PGS_NT_) polygenic risk scores, as well as half nontransmitted polygenic scores separated by parent analyzed in a joint model.

## Data Availability

Data may be obtained from a third party and are not publicly available. Researchers can apply to use the Lifelines data used in this study. More information about how to request Lifelines data and the conditions of use can be found on their website (https://www.lifelines.nl/researcher/how-to-apply).
