# Supplementary material for: Using parent-offspring pairs and trios to estimate indirect genetic effects in education
Source: Genet Epidemiol. Author manuscript; Available in PMC 2024 Aug 23. (PMC11343084; doi:10.1002/gepi.22554)
Supplement: Supplement [file NIHMS2010921-supplement-Supplement.docx]

Using parent-offspring pairs and trios to estimate indirect genetic effects in education:

Supplementary Materials

1. Models

We fitted cumulative link mixed models for the secondary school level and educational attainment outcomes and a linear mixed model for years of education. For each outcome Y in individual *i* in family *j*, the model can be expressed as follows:

$Yij \sim\left( 1 \right| FIDj)+ \beta PGST+\beta PGSNT+ \beta sex+\beta age+ \beta PC1...\beta PC10+\varepsilon$

$\beta PGST$ represents the effect of the offspring's own (hence, transmitted) PGS on the outcome, while $\beta PGSNT$ represents the joint indirect effects of parental genotypes (βindirect). $FIDj$ is a factor variable that groups siblings together based on their family identifier. Both PGS_T_ and PGS_NT_ capture IGEs, given that it is expected that some of the transmitted variants are also associated with the environmental exposures or parental characteristics that influence the offspring's outcomes. Therefore, direct genetic effects can be estimated as βdirect = $\beta PGST$ - $\beta PGSNT$.

To compare our estimated IGEs to previous studies, we calculated the ratio of indirect to direct genetic effects $\frac{\beta indirect}{\beta direct}$.

For parent-of-origin analyses, the PGS_NT_ was split in paternal (PGS_NTp_) and maternal (PGS_NTm_) and both were included as predictors in the same model. Additionaly, we tested the associations of PGS_NTp_ and PGS_NTm_ with the outcomes in separate models:

$Yij\sim\left( 1 \right| FIDj)+ \beta PGST+\beta PGSNTm+ \beta sex+\beta age+ \beta PC1...PC10+\varepsilon$

$Yij\sim\left( 1 \right| FIDj)+ \beta PGST+\beta PGSNTp+ \beta sex+\beta age+ \beta PC1...PC10+\varepsilon$

In these analyses, we standardized PGS_NTp_ and PGS_NTm_ so that each would have SD = 1 and variance = 0.5, by dividing by the standard deviation used to standardize the full PGS_T_:

Z= $\frac{x-mean (PGSNTp)}{SD(PGST)}$.

We performed Brant tests to evaluate the proportional odds assumption in the ordinal models presented in this study. This test compares a generalized ordinal logistic regression model with a proportional odds model by assessing if coefficient differences are significant with a chi-square statistic (Brant, 1990). Results support that the proportional odds assumption for the main predictors in is valid in both secondary school level (PGS_T_ χ²= 0.96, df=1, p=0.33; PGS_NT_ χ²=0.25, df=1, p=0.62) and educational attainment (PGS_T_ χ² = 0.03, df=1, p=0.86; PGS_NT_ χ²=0.04, df=1, p=0.085) models.

1. SNP density

We found that there are slight more discrepancies between our method and PseudoCons in chromosome 6. This is because more markers are measured in a the multiple histocompatibility complex region in the array used in this study (Infinium Global Screening Array). This means the measured markers around that region will be closer in location to each other and more likely to be in linkage disequilibrium (correlated). If too many linked markers are included in each tile, there is less specificity in the match between parental and offspring haplotypes, as the markers will not be independent and thus will not allow for distinction of possible recombination spots. In conclusion, the number of markers to be included in a tile depends on the SNP density measured by the array. We decided to remove markers in this specific region from the PGS.

Tables

Supplementary Table 1. Coding of secondary school level variable according to highest obtained degree for participants aged >18 in youth sample.

| **Highest obtained degree** | **Secondary school level** |
| --- | --- |
| VMBO or  preparatory secondary vocational education | 1 (VMBO) |
| HAVO or  higher vocational education | 2 (HAVO) |
| VWO or research university education | 3 (VWO) |

|  | | **Paternal only** | | | **Maternal only** | | |
| --- | --- | --- | --- | --- | --- | --- | --- |
|  | | Beta (SD) | 95% CI | p | Beta (SD) | 95% CI | p |
| **Youth** |  | N=930 | | | N=1145 | | |
|  | **Secondary school** |  | | |  | | |
|  | PGS_T_ | 0.41 (0.06) | 0.27 - 0.54 | <0.001 | 0.47 (0.06) | 0.34 - 0.60 | <0.001 |
|  | PGS_NT_ | 0.08 (0.09) | -0.10 - 0.27 | 0.36 | 0.20 (0.09) | 0.02 - 0.38 | 0.03 |
| **Adults** | | N= 2032 | | | N=2793 | | |
|  | **Educational attainment** |  | | |  | | |
|  | PGS_T_ | 0.55 (0.05) | 0.44 – 0.65 | <0.001 | 0.55 (0.04) | 0.46 – 0.64 | <0.001 |
|  | PGS_NT_ | 0.15 (0.07) | 0.01 – 0.29 | 0.03 | 0.25 (0.06) | 0.13 – 0.37 | <0.001 |
|  |  | | | | | | |
|  | **Years of education** |  | | | | | |
|  | PGS_T_ | 0.90 (0.08) | 0.74 – 1.06 | <0.001 | 0.94 (0.07) | 0.79 – 1.08 | <0.001 |
|  | PGS_NT_ | 0.27 (0.12) | 0.05 – 0.50 | 0.02 | 0.41 (0.10) | 0.20 – 0.61 | <0.001 |

Supplementary Table 2. Parent-of-origin sensitivity analyses.

The effect sizes are shown for separate models using only the non-transmitted polygenic score (PGS_NT_) for fathers or mothers. These models do not use mean imputation for the half PGS_NT_ for the missing parent. In these separate models, each educational outcome is regressed separately on the PGS_NT_ for non-missing mothers or fathers with covariates. This is in contrast to joint models which include both PGS_NT_ for mothers and fathers in the same model. Effect sizes are reported with standardized betas, along with their standard deviation (SD) and 95% confidence intervals (CI).

Supplementary Figure 1. Power analysis for non-transmitted polygenic scores for educational attainment (PGS_NT_) based on simulations.


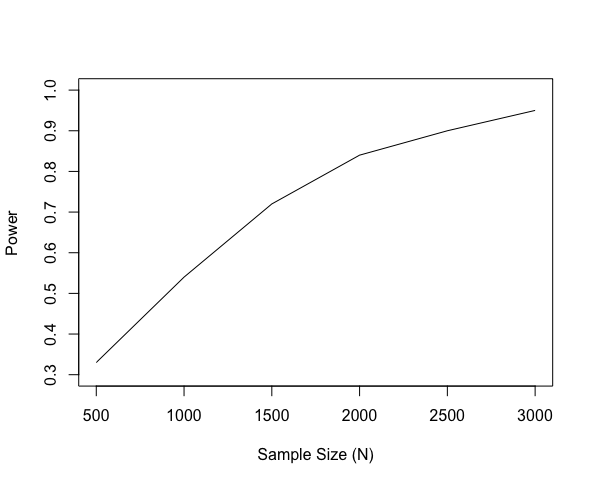


The analysis was conducted to determine the sample size needed to detect the effect size for PGS_NT_ for educational attainment in the adult subsample (beta = 0.17). The original dataset was resampled using a bootstrapping technique, generating 10,000 datasets for each sample size tested from 500 to 3000. The proportion of resampled datasets in which the null hypothesis was rejected was used to estimate the power. Results show that the sample of 561 trios would have low statistical power, but using our haplotype-based approach that added parent-offspring pairs allowed us to increase the sample size by around 7.6-fold (N=4,264), which improved the statistical power to detect the indirect genetic effects.
